# Heart Rate and Respiration Affect the Functional Connectivity of Default Mode Network in Resting-State Functional Magnetic Resonance Imaging

**DOI:** 10.3389/fnins.2020.00631

**Published:** 2020-06-30

**Authors:** Akira Yoshikawa, Yuri Masaoka, Masaki Yoshida, Nobuyoshi Koiwa, Motoyasu Honma, Keiko Watanabe, Satomi Kubota, Iizuka Natsuko, Masahiro Ida, Masahiko Izumizaki

**Affiliations:** ^1^Department of Physiology, School of Medicine, Showa University, Tokyo, Japan; ^2^School of Nursing and Rehabilitation Sciences, Showa University, Yokohama, Japan; ^3^Department of Ophthalmology, The Jikei University School of Medicine, Tokyo, Japan; ^4^Department of Health and Science, University of Human Arts and Sciences, Hasuda, Japan; ^5^Department of Neurology, School of Medicine, Showa University, Tokyo, Japan; ^6^National Hospital Organization Mito Medical Center, Mito, Japan

**Keywords:** resting-state functional magnetic resonance imaging, default mode network, physiological noise, respiration, cardiac output

## Abstract

A growing number of brain imaging studies show functional connectivity (FC) between regions during emotional and cognitive tasks in humans. However, emotions are accompanied by changes in physiological parameters such as heart rate and respiration. These changes may affect blood oxygen level-dependent signals, as well as connectivity between brain areas. This study aimed to clarify the effects of physiological noise on the connectivity between areas related to the default mode network using resting-state functional magnetic resonance imaging (rs-fMRI). Healthy adult volunteers (age range: 19–51 years, mean age: 26.9 ± 9.1 years, 8 males and 8 females) underwent rs-fMRI for 10 min using a clinical 3T scanner (MAGNETOM Trio A Tim System, Siemens) with simultaneously recorded respiration and cardiac output. Physiological noise signals were subsequently removed from the acquired fMRI data using the DRIFTER toolbox. Image processing and analysis of the FC between areas related to the default mode network were performed using DPARSF. Network-Based Statistic (NBS) analysis of the functional connectome of the DMN and DMN-related area was used to perform three groups of comparison: without physiological noise correction, with cardiac noise correction, and with cardiac and respiratory noise correction. NBS analysis identified 36 networks with significant differences in three conditions in FC matrices. *Post hoc* comparison showed no differences between the three conditions, indicating that all three had the same networks. Among the 36 networks, strength of FC of 8 networks was modified under physiological noise correction. Connectivity between left and right anterior medial frontal regions increased strength of connectivity. These areas are located on the medial cerebral hemisphere, close to the sagittal sinus and arteries in the cerebral hemispheres, suggesting that medial frontal areas may be sensitive to cardiac rhythm close to arteries. The other networks observed temporal regions and showed a decrease in their connectivity strength by removing physiological noise, indicating that physiological noise, especially respiration, may be sensitive to BOLD signal in the temporal regions during resting state. Temporal lobe was highly correlated with anxiety-related respiration changes (Masaoka and Homma, 2000), speech processing, and respiratory sensation. These factors may affect the rs-fMRI signaling sensitivity.

## Introduction

Neuroimaging studies in humans have identified key areas for various emotions, as well as functional connectivity (FC) between brain regions (Maddock et al., 2003; Greicius et al., 2007; Zhi et al., 2018). Resting-state functional magnetic resonance imaging (rs-fMRI) is suitable for studying brain functions, particularly the default mode network (DMN), which is related to emotional and cognitive control (Raichle et al., 2001; Raichle, 2015; Tang et al., 2015). Emotions, especially negative emotions such as fear and anxiety (Homma and Masaoka, 2008; Masaoka et al., 2014b), are accompanied by physiological changes in parameters, including heart rate, respiration, and skin conductance (Boiten et al., 1994; Masaoka et al., 2014b; Mather and Thayer, 2018). Functional neuroimaging studies have focused on how negative emotions are inhibited, for example, when participants perform mindfulness tasks concentrating on breathing cycles (Tang et al., 2015). Indeed, slower breathing can modulate the anxiety level, which is reflected by changes in amygdala activation, as well as the connectivity between the amygdala and prefrontal areas (Banks et al., 2007; Kim et al., 2011; Doll et al., 2016). Various reports have indicated that mindfulness actually results in slower breathing cycles with increased DMN activation (Tang et al., 2015; Doll et al., 2016), which is involved in mind wandering (Mason et al., 2007; Kucyi et al., 2013; Taruffi et al., 2017), self-referential processing (Northoff et al., 2006; Sheline et al., 2009; van Buuren et al., 2010), and memory retrieval (Sestieri et al., 2011; Xu et al., 2014). Strengthening of the FC between the DMN areas has been shown to improve mindfulness or/and self-cognitive skills (Brewer et al., 2011; Hasenkamp and Barsalou, 2012), and these effects might be associated with changes in the physiological state. Although these neuroimaging studies present evidence for the benefits of these tasks in our daily life, how these physiological changes affect blood oxygen level-dependent (BOLD) signals and brain region connectivity is unknown. Considering that the DMN involves the medial parts of the brain such as the medial prefrontal, anterior cingulate, and posterior cingulate cortices (McKiernan et al., 2003; Greicius et al., 2009), which are near the sagittal sinus and arteries, we hypothesized that these brain regions may be affected by respiration and cardiac output. In this study, we simultaneously measured cardiac and respiratory output parameters on rs-fMRI to address the following questions: (1) Which areas of the network are most likely to be influenced by cardiac and respiratory rhythms? (2) How do the respiratory rate (RR) and pulse rate (PR) affect the BOLD signals and DMN?

## Materials and Methods

### Participants

A total of 16 healthy volunteers were selected for this study (age range: 19–51 years, mean age: 26.9 ± 9.1 years, 8 males and 8 females; Table 1). This study was reviewed and approved by the Ethics Committee of Showa University School of Medicine. All participants provided written informed consent prior to the experiment.

**TABLE 1:** Demographics, respiratory rate (RR), and pulse rate (PR) of the study participants and their statistical comparison.

		Number	Mean	Std. deviation	Std. error mean	*t*	Degree of freedom	Sig. (2-tailed)
Age	F	8	24.4	6.6	2.3	1.10	14	0.288
	M	8	29.4	11.0	3.9			
	Total	16	26.9	9.1	2.3			
RR	F	8	15.4	4.6	1.6	−1.66	14	0.120
	M	8	11.4	5.0	1.8			
	Total	16	13.4	5.1	1.3			
PR	F	8	68.7	18.3	6.5	−0.33	14	0.758
	M	8	66.2	11.5	4.1			
	Total	16	67.4	14.8	3.7			

### fMRI Data Acquisition

MRI scanning was performed at Ebara Hospital (Tokyo, Japan) using a 3T MAGNETOM Trio A Tim scanner (Siemens, Erlangen, Germany). For 600-s rs-fMRI scanning session, participants were instructed to lie with their eyes closed and to intensively think about anything that they wanted. Six hundred contiguous whole-brain T2^∗^-weighted echo-planar images were acquired using a 32-channel phased-array head coil. To increase the temporal resolution, functional imaging consisted of multiband accelerated gradient-echo echo-planar imaging that excited four slices simultaneously (multiband = 4). The sequence parameters were as follows: repetition time = 1 s; echo time = 27 ms; field of view = 200 mm; matrix = 80 × 80; in-plane resolution equal to 2.5 mm × 2.5 mm, 39 slices; thickness = 2.5 mm, producing isometric voxels. Anatomical scan was acquired with a T1-weighted 3D MPRAGE sequence: 9° flip angle; repetition time = 2,300 ms; echo time = 2.98 ms; matrix size 256 × 256; field of view = 256 mm; 176 slices with a voxel size of 1 mm^3^.

### Physiological Data Acquisition

**FIGURE 1:**
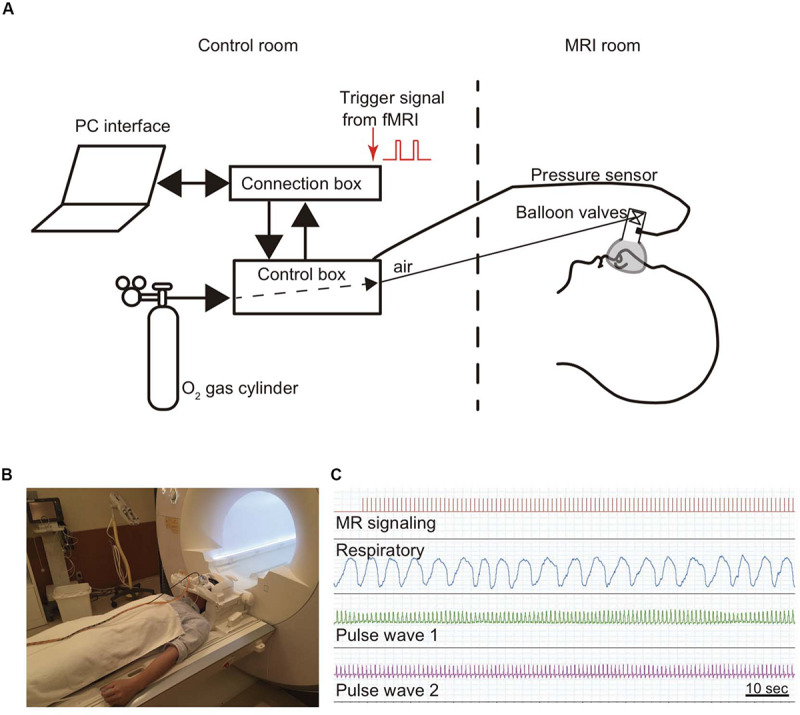
Method for monitoring respiratory and pulse rates. A modified olfactory stimulator was used when monitoring respiration (Masaoka et al., 2014a). **(A)** Briefly, the participant wears a nose mask with a pressure sensor and one-way valve apparatus in the scanner. Airflow is regulated using compressed O_2_ and sent from the control room via a urethane tube. The respiratory rate is calculated from the pressure signals. **(B)** Photograph of a participant wearing a nose mask in the MRI scanner room. **(C)** The pressure signal with the inspiratory and expiratory flow, which is converted from an analog to a digital signal, is sent via a control box to the connection box and stored together with the cardiac output and the fMRI signals in LabChart through PowerLab.

The participants were instructed to breathe normally through a nose mask (Figures 1A,B). This mask (ComfortGel Blue Nasal Mask 1070038, medium size; Phillips Respironics, Murrysville, PA, United States) was designed to measure the respiratory flow, and a piezoelectric pressure transducer was attached because the RR was measured by a urethane tube. This nose mask was also fitted with a one-way valve apparatus to ensure the inspiration of air from the control box and expiration out of the system. The control box was MRI-compatible (ARCO System, Chiba, Japan; Figure 1A). The PR was recorded from both first toes using a photoplethysmogram transducer (TSD200-MRI and PPG100C-MRI; Bio Pac, LA System, Japan). The pressure signals of both blood volume pulse waveform and inspiratory–expiratory flow were converted from an analog to a digital signal and stored in a control box using LabChart through PowerLab (ML846; ADInstruments, Aichi, Japan; Figures 1A,C). Further details about this method for monitoring RR and PR have been described previously (Masaoka et al., 2014a; Watanabe et al., 2018).

### Physiological Signal Preprocessing

Signal preprocessing was performed using statistical parametric mapping (SPM8 and SPM12) software (Wellcome Department of Cognitive Neurology, London, United Kingdom) implemented in MATLAB (R2015B; MathWorks Inc., Natick, MA, United States) on a computer running OS X El Capitan. The RETROICO (Glover et al., 2000) or DRIFTER (Sarkka et al., 2012) toolboxes have been used as tools for removing physiological noises. We employed the DRIFTER algorithm (Sarkka et al., 2012), which is a Bayesian method, to determine the physiological noise and separate it from the fMRI signal. Respiration and heart rate are phenomena that change every minute; respiration changes even from breath to breath. DRIFTER can track the changes in both amplitude and shape in the periodic noise and separate the physiological noise from the acquired fMRI data. Based on its characteristics, we considered using DRIFTER beneficial to remove physiological noise without changes in the reference signal (Rajna et al., 2015). The temporal dynamics of the frequency set in DRIFTER for the cardiac and respiratory noise were estimated from the fMRI signals.

### Image Processing and Analysis

The images were preprocessed as follows using the software DPARSF^1^ (Chao-Gan and Yu-Feng, 2010; Yan et al., 2016) and SPM12^2^ : differences in image acquisition time between slices were corrected; the time series of images were realigned to remove movement artifacts; the images were normalized to a standard SPM8 EPI template, which warps each individual subject into a standard space with a resolution of 3 mm × 3 mm × 3 mm based on the Montreal Neurological Institute (MNI) template; and the images were smoothed with full width at half maximum (FWHM) specified as 4 mm. Detrending and band-pass filtering (0.01–0.1 Hz) of the BOLD signals were performed to remove both low-frequency drift and high-frequency noise. Additionally, interferences were regressed out, which included the head motion parameters, white matter signals, cerebrospinal fluid signals, and global mean signals. Regarding head movement, it has been suggested that FC in short distances increases, while FC in long distances decreases (Power et al., 2012). In DPARSF, head movement parameters were computed in each direction (*x*, *y*, *z*) along with angular rotation on each axis (pitch, roll, and yaw) (Friston et al., 1996). In this study, we decided to remove the frames showing Δ 3 mm of head movement in one frame to the next (Gaudio et al., 2018). To evaluate the association between head movement and physiological noise, correlation analysis and multiple regression with interaction analyses were conducted. Each head movement data, which is the average value of 600 scans by corrected Drif_NO, Drif_C, or Drif_CR data, was the dependent variables in each regression model, while the PR and RR were entered as independent variables. To test for differences in slopes for PR/RR against head movement between Drif_NO and Drif_C or Drif_CR, we used dummy variables to evaluate whether the slopes of Drif_C and Drif_CR differed from the slope of Drif_NO.

### Data Analysis

The functional brain images of each subject were mapped to the automated anatomical labeling (AAL) brain template (Tzourio-Mazoyer et al., 2002), which is commonly used with NBS (Zalesky et al., 2010b). The AAL areas included the DMN and DMN-related brain regions—namely, the bilateral superior medial frontal gyrus, superior medial orbital frontal gyrus, anterior cingulate gyri, median cingulate gyri, posterior cingulate gyri, superior parietal gyrus, inferior parietal gyrus, supramarginal gyrus, angular gyrus, precuneus, paracentral lobule, superior temporal gyrus, middle temporal gyrus, and inferior temporal gyrus (Supplementary Table S1). Using DPARSF, we obtained the averaged time courses from each region of interest in the defined DMN and DMN-related brain areas; the Pearson correlation coefficient *r* for the analysis of the BOLD time course within each region of interest in the DMN and DMN-related brain areas was also automatically obtained by DPARSF. For statistical analysis, *z*-value, obtained by Fisher’s *r*-to-*z* transformation, was used; this Fisher’s *r*-to-*z* transformation was also calculated automatically using DPARSF software. Network-Based Statistic (NBS)^3^ (Zalesky et al., 2010a) analysis was used to compare the three conditions. For each of the FC formed between nodes in the network composed in DMN and DMN-related areas, multi-statistical analysis was performed using *z*-value in the three conditions. In this NBS, threshold and significant *p*-value were 4.0 and <0.01, respectively. Subsequent NBS analysis was performed using *t*-test in each Drif_NO vs. Drif_C, Drif_NO vs. Drif_CR, and Dric_C vs. Drif_CR. In order to investigate the FC strength within each subnetwork identified by NBS analysis, paired *t*-test was performed to compare the FC survived with NBS in the three conditions using SPSS version 25 (IBM Corp., Armonk, NY, United States). To correct for multiple comparisons, a false discovery rate (FDR) procedure was performed at *q* < 0.05 (Zhan et al., 2019).

Following statistical analysis of the association between FC and physiological noise, multiple regression with interaction analysis was performed to compare slopes between rs-fMRI data that was Drif_NO, Drif_C, and Drif_CR in order to assess whether differential relationships exist between RR, PR, and *z* scores, with the *z* scores being the dependent variables in each regression model. For independent variables, we entered each physiological rate, condition, and all interactions (e.g., PR × Drif_C). To test for differences in slopes for PR/RR against *z* scores between Drif_NO and Drif_C or Drif_CR, we used dummy variables to evaluate whether the slopes of Drif_C and Drif_CR differed from the slope of Drif_NO.

For exploratory analysis, BOLD signals (mean, 600 s) were extracted from several nodes that showed significant difference in the FC strength analysis from each subject to compare the three conditions with one-way ANOVA. All statistical analyses were performed using SPSS version 25 (IBM Corp., Armonk, NY, United States).

## Results

### Physiological Data

During rs-fMRI scanning, PR and RR were recorded. The mean RR and PR values were 13.4 ± 5.1/min and 67.4 ± 14.8/min, respectively. There were no statistically significant differences in these physiological data between males and females (Table 1).

### The Relationship Between Head Movement and Physiological Data

**FIGURE 2:**
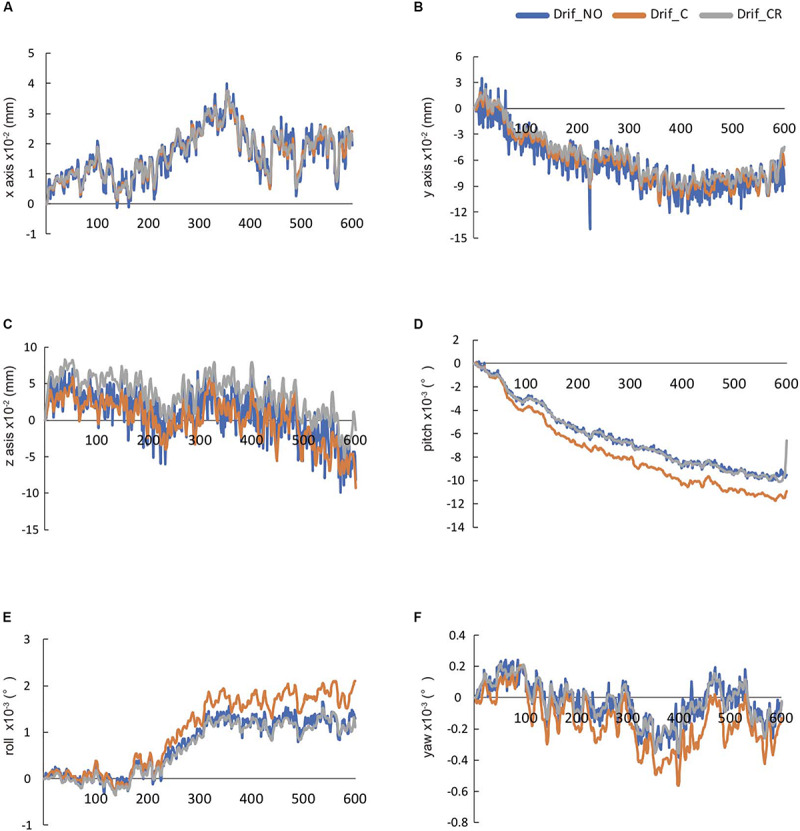
The temporal changes in 600-s head movement in raw data. These data were automatically calculated by DPARSF. The head movement in *x*-axis **(A)**, *y*-axis **(B)**, and *z*-axis **(C)**, respectively. The head rotation movement of pitch in *x*-axis **(D)**, roll in *y*-axis **(E)**, and yaw in *z*-axis **(F)**, respectively.

**FIGURE 3:**
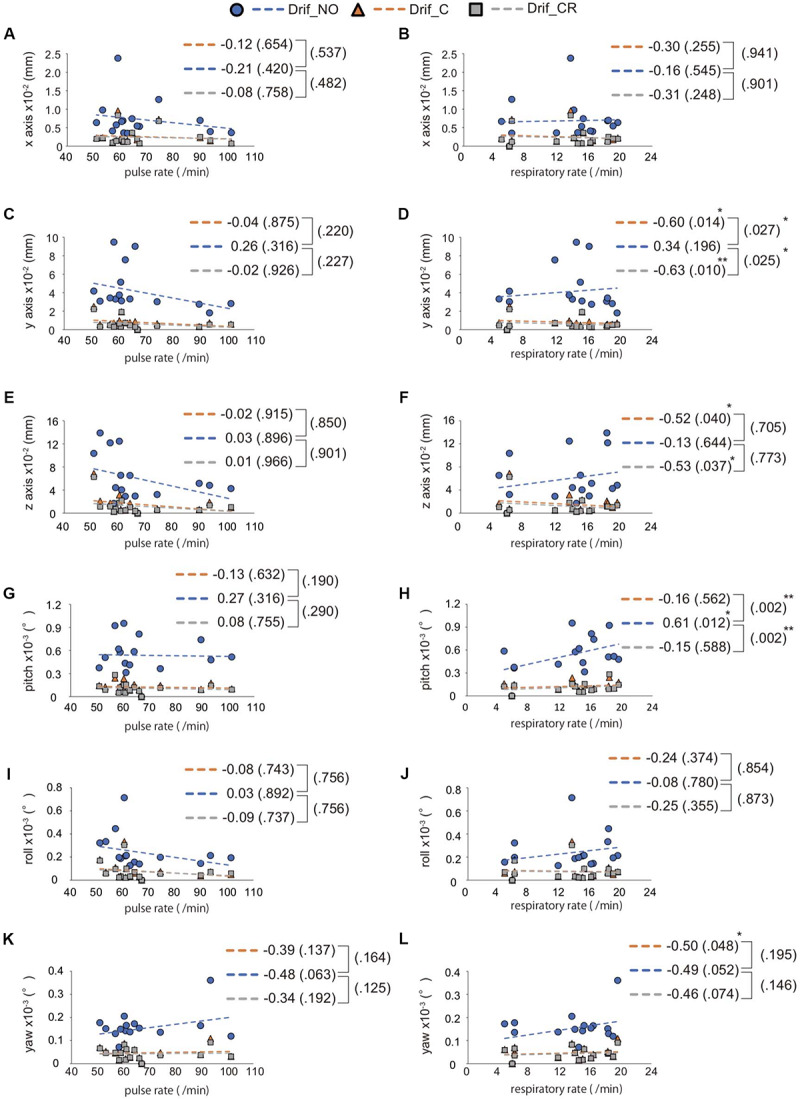
Interaction between RR/PR and the average of head movement. The left side image **(A,C,E,G,I,K)** indicates the relationship between pulse rate (PR) and head movement, whereas the right side image **(B,D,F,H,J,L)** indicates the relationship between respiratory rate (RR) and head movement. The direction of head movement is indicated on the *x*-axis **(A,B)**, *y*-axis **(C,D)**, and *z*-axis **(E,F)**. The rotation of head movement indicates pitch **(G,H)**, roll **(I,J)**, and yaw **(K,L)**. The correlation coefficient is shown in the side of each broken line and *p*-value for correlation coefficient is shown in parentheses. The result of interaction between Drif_NO and Drif_C or Drif_CR is indicated on the right side of the single bracket as a *p*-value in parentheses. There is no significant correlation coefficient and interaction between head movement and PR but significant difference is shown in correlation coefficient and interaction between head movement and RR. ^∗^*p* < 0.05, ^∗∗^*p* < 0.01, with statistical analysis. See Table 2 for the distance of head movement and the angle of head rotation.

We evaluated the head movement in each direction (*x*, *y*, *z*) and angular rotation on each axis (pitch, roll, and yaw) during the 600 scanning since head movement is one of the most common sources of noise to affect FC (Figure 2). There was a significant difference in all head movement, whereas no maximum head movement that was more than 3 mm movement was found (Table 2). It was indicated that the correction of physiological noise using DRIFTER algorithm corrected the head movement under our localized head movement. Under the limitation of head movement, the relationship between the PR/RR and the head movement was investigated (Figure 3). No significant difference between PR and head movement or interaction in three conditions was shown (Figures 3A,C,D,G,I,K). Conversely, RR had an influence on the head movement. There was no significant difference in the *x*-axis (Figure 3B), whereas in the *y*-axis, it was shown that the physiological noise correction significantly restricted the head movement as RR increased. Moreover, Drif_NO tended to increase the head movement and significant interactions between Drif_NO and Drif_C and between Drif_NO and Drif_CR were indicated (Figure 3D). In the *z*-axis, the negative significant correlation between the increasing of head movement and increasing RR was indicated under the condition of physiological noise correction but no significant interaction was found (Figure 3F). In the head movement of pitch, the positive significant correlation between the increasing of angle, as pitch, and the increasing RR was shown but no interaction was found (Figure 3H). There was no significant correlation and interaction in the head movement of roll (Figure 3J). In the head movement of yaw, negative significant correlation between the increasing of angle, as yaw, and the increasing RR was indicated but no interaction was found (Figure 3L).

**TABLE 2:** The average of the head movement in each direction (*x*, *y*, *z*) and angular rotation on each axis (pitch, roll, and yaw) during 600 scanning.

	Drif_NO	Drif_C	Drif_CR	Sig. (*p*)
*x*-axis: mm (×10^–2^)	0.72 ± 0.50	0.26 ± 0.24	0.24 ± 0.22	0.001*
				0.001*
Max value	5.36 ± 3.39	1.45 ± 1.13	2.70 ± 1.95	1.000
*y*-axis: mm (×10^–2^)	4.34 ± 2.29	0.85 ± 0.57	0.64 ± 0.57	<0.001*
				<0.001*
Max value	18.62 ± 16.39	4.52 ± 2.36	4.64 ± 3.52	
				1.000
*z*-axis: mm (×10^–2^)	6.15 ± 3.89	1.59 ± 1.63	1.26 ± 1.44	<0.001*
				<0.001*
Max value	28.97 ± 18.13	7.67 ± 5.18	8.62 ± 7.48	
				1.000*
Pitch: degree (×10^–3^)	0.57 ± 0.19	0.13 ± 0.05	0.11 ± 0.06	<0.001*
				<0.001*
Max value	2.95 ± 1.34	0.83 ± 0.38	1.81 ± 2.52	
				1.000
Roll: degree (×10^–3^)	0.25 ± 0.15	0.08 ± 0.08	0.07 ± 0.07	<0.001*
				<0.001*
Max value	1.79 ± 1.41	0.46 ± 0.33	1.08 ± 0.73	
				1.000
Yaw: degree (×10^–3^)	0.16 ± 0.06	0.05 ± 0.03	0.04 ± 0.02	<0.001*
				<0.001*
Max value	1.11 ± 0.47	0.29 ± 0.15	0.41 ± 0.37	
				1.000

**In Sig (p) column, the value in the upper, middle, and lower row indicates Drif_NO vs. Drif_C, Drif_NO vs. Drif_CR, and Drif_C vs. Drif_CR, respectively. ^∗^indicates significant difference by Bonferroni post hoc analysis*.*

### NBS Analysis in the DMN and DMN-Related Brain Regions With and Without Physiological Noise Correction

**FIGURE 4:**
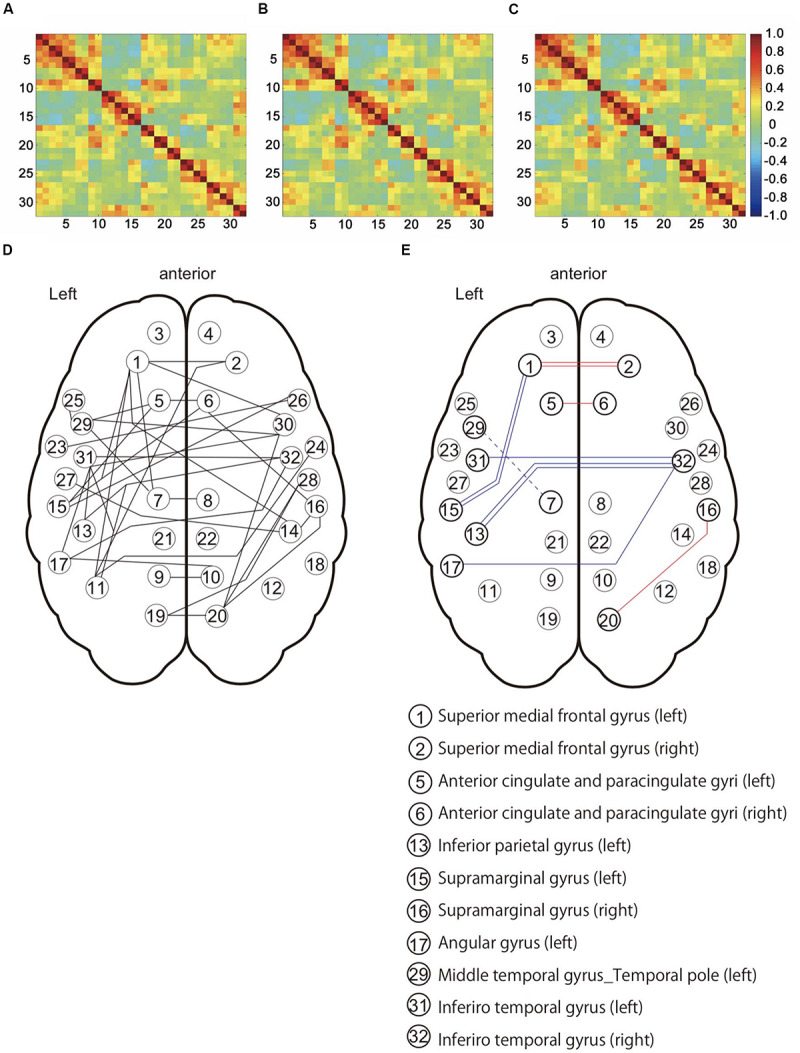
Functional connectivity patterns among the default mode network (DMN) and DMN-related brain regions in 16 subjects. **(A)** Connectivity matrix with no corrected physiological noise (Drif_NO). **(B)** Connectivity matrix corrected for cardiac noise (Drif_C). **(C)** Connectivity matrix corrected for cardiac and respiratory noise signals (Drif_CR). The value of these three matrixes is the correlation coefficient (*r*). **(D)** This network showed significant difference in the three conditions (Drif_NO, Drif_C, and Drif_CR) using Network-Based Statistic (NBS) (Zalesky et al., 2010a). **(E)** Results of FC strength with significant difference. Following NBS analysis, paired *t*-test and *post hoc* false discovery rate (FDR) correction was performed so as to further evaluate the strength of individual FC. The number of 13 nodes and 8 FC had significant differences. The red line indicates an increase in FC strength, while the blue line indicates a decrease in FC strength compared to no physiological noise correction. The single red line indicates that FC of Drif_C was significantly increased compared to Drif_NO. The double red line indicates that FC of both Drif_C and Drif_CR was significantly increased compared to Drif_NO. The single blue line indicates that FC of Drif_C was significantly decreased compared to Drif_NO. The double blue line indicates that FC of both Drif_C and Drif_CR was significantly decreased compared to Drif_NO. The broken blue line indicates that FC of Drif_CR was significantly decreased compared to Drif_NO. See Table 3 for the *z*-value in each strength of FC.

We evaluated the influence in the DMN and DMN-related brain region under the three preprocessing conditions (Figures 4A–C). NBS analysis identified 36 networks with significant differences in FC matrices (Figure 4D). Paired *t*-test was performed to compare the three groups’ networks, and there was no significant network difference in the three conditions. To investigate the FC strength in the three groups, paired *t*-tests and *post hoc* FDR correction tests were performed in 36 connections, and 8 FC made by13 nodes, which showed significant differences (Table 3) (Figure 4E). For details on the eight FC, FC strength was increased in three networks (Figure 4E: red line), while another five networks decreased FC strength (Figure 4E: blue line). In three networks (Figure 4E: red line), FC strength was increased between the left and right superior medial frontal gyrus, between the left and right anterior cingulate gyrus, and between the right supramarginal gyrus and the right precuneus. In another five networks (Figure 4E: blue line), FC strength was decreased: between the left and right inferior temporal gyrus, between the right inferior temporal gyrus and the left inferior parietal gyrus, between the right inferior temporal gyrus and the left angular gyrus, between the left superior medial frontal gyrus and the left supramarginal gyrus, and between the left median cingulate gyri and the left middle cingulate gyrus.

**TABLE 3:** Results of the NBS and *post hoc* FDR analysis.

Connectivity	Drif_NO	Drif_C	Drif_CR	Sig. (*p*)
Superior medial frontal gyrus (left) to superior medial frontal gyrus (right)	0.950 ± 0.24	1.103 ± 0.20	1.085 ± 0.21	0.001* 0.004* 0.367
Anterior cingulate and paracingulate gyri (left) to anterior cingulate and paracingulate gyri (right)	1.236 ± 0.22	1.386 ± 0.24	1.351 ± 0.20	0.005* 0.021 0.127
Superior medial frontal gyrus (left) to supramarginal gyrus (left)	−0.135 ± 0.25	−0.173 ± 0.29	−0.242 ± 0.27	0.236* 0.001* 0.002
Supramarginal gyrus (right) to precuneus (right)	−0.081 ± 0.24	0.031 ± 0.20	0.020 ± 0.19	0.003 0.026* 0.496
Median cingulate and paracingulate gyri (left) to middle temporal gyrus_Temporal pole (left)	0.001 ± 0.30	−0.051 ± 0.32	−0.085 ± 0.33	0.079 0.002 0.164*
Inferior parietal gyrus (left) to inferior temporal gyrus (right)	0.527 ± 0.24	0.370 ± 0.30	0.393 ± 0.26	0.002* 0.004* 0.258
Angular gyrus (left) to inferior temporal gyrus (right)	0.355 ± 0.23	0.226 ± 0.24	0.252 ± 0.21	0.007* 0.024 0.369
Inferior temporal gyrus (left) to inferior temporal gyrus (right)	0.865 ± 0.33	0.679 ± 0.42	0.714 ± 0.37	<0.001* 0.019 0.473
				

**In Sig (p) column, the values in the upper, middle, and lower row indicate Drif_NO vs. Drif_C, Drif_NO vs. Drif_CR, and Drif_C vs. Drif_CR, respectively. ^∗^indicates FDR < 0.05. Average ± SD*.*

### Influence of the Physiological Noise on BOLD Signals

Prior to investigating the influence of PR or RR on the eight FC, we evaluated the temporal changes in 600-s BOLD signals from both sides of the superior medial frontal gyri, both sides of the anterior cingulate gyri, left median cingulate gyri, left inferior parietal gyrus, left supramarginal gyrus, right supramarginal gyrus, left angular, right precuneus, left middle temporal gyrus_temporal pole, left inferior temporal gyrus, and right inferior temporal gyrus that were nodes with significantly differences (Supplementary Figure S3). BOLD signal in these nodes was automatically calculated by DPARSF software and statistically analyzed between three conditions—namely, Drif_NO, Drif_C, and Drif_CR. The average of 600-s BOLD signals showed no significant differences between the three conditions although the head movement was significantly different between the three conditions (Supplementary Table S2). It has been reported that head movement affects these BOLD signals (Power et al., 2012), but the reason why no significant difference was observed is that the head movements were limited (Friston et al., 1996).

### Interaction Between RR/PR and Condition

To investigate the influence of PR or RR on the FC with significant differences in FDR correction analysis, individual correlations between the *z*-value and PR or RR were examined for the three defined conditions (Drif_NO, Drif_C, and Drif_CR). There was no significant difference between PR and *z*-value in all conditions; conversely, one significant difference was shown between RR and *z*-value in the FC made by the left inferior parietal gyrus and the right inferior temporal gyrus in the three conditions. In all FC, there was no significant interaction between Drif_C and Drif_NO and between Drif_CR and Drif_NO for the relationship between PR and *z*-value and between RR and *z*-value (Table 4).

**TABLE 4:** Results of the multiple regression and interaction analysis.

		Pulse rate (PR)			Respiratory rate (RR)		
		Drif_NO	Drif_C	Drif_CR	Drif_NO	Drif_C	Drif_CR
Superior medial frontal gyrus (left) to superior medial frontal gyrus (right)	*r*	0.26	0.27	0.40	0.31	0.15	0.21
	(Sig.)	(0.333)	(0.319)	(0.127)	(0.24)	(0.59)	(0.44)
	Drif_NO - Drif_C (sig.)	0.914			0.573		
	Drif_NO - Drif_CR (sig.)	0.766			0.704		
Anterior cingulate and paracingulate gyri (left) to anterior cingulate and paracingulate gyri (right)	*r*	0.08	0.16	0.38	0.25	0.26	0.28
	(Sig.)	(0.775)	(0.553)	(0.145)	(0.359)	(0.339)	(0.297)
	Drif_NO - Drif_C (sig.)	0.791			0.922		
	Drif_NO - Drif_CR (sig.)	0.471			0.983		
Superior medial frontal gyrus (left) to supramarginal gyrus (left)	*r*	−0.06	0.08	0.09	0.29	0.31	0.30
	(Sig.)	(0.811)	(0.762)	(0.745)	(0.284)	(0.241)	(0.255)
	Drif_NO - Drif_C (sig.)	0.791			0.922		
	Drif_NO - Drif_CR (sig.)	0.471			0.983		
Supramarginal gyrus (right) to precuneus (right)	*r*	0.13	−0.11	−0.18	0.37	0.18	0.07
	(Sig.)	(0.644)	(0.693)	(0.496)	(0.153)	(0.510)	(0.809)
	Drif_NO - Drif_C (sig.)	0.517			0.472		
	Drif_NO - Drif_CR (sig.)	0.415			0.312		
Median cingulate and paracingulate gyri (left) to middle temporal gyrus_Temporal pole (left)	*r*	−0.10	0.00	−0.08	0.31	0.28	0.20
	(Sig.)	(0.713)	(0.995)	(0.771)	(0.246)	(0.294)	(0.447)
	Drif_NO - Drif_C (sig.)	0.805			0.966		
	Drif_NO - Drif_CR (sig.)	0.974			0.829		
Inferior parietal gyrus (left) to inferior temporal gyrus (right)	*r*	0.35	0.23	0.27	0.59*	0.64**	0.71**
	(Sig.)	(0.180)	(0.400)	(0.311)	(0.017)	(0.007)	(0.002)
	Drif_NO - Drif_C (sig.)	0.859			0.513		
	Drif_NO - Drif_CR (sig.)	0.885			0.557		
Angular gyrus (left) to inferior temporal gyrus (right)	*r*	0.00	0.01	0.07	−0.02	0.14	0.20
	(Sig.)	(0.992)	(0.975)	(0.794)	(0.930)	(0.603)	(0.454)
	Drif_NO - Drif_C (sig.)	0.975			0.649		
	Drif_NO - Drif_CR (sig.)	0.857			0.576		
Inferior temporal gyrus (left) to inferior temporal gyrus (right)	*r*	−0.01	−0.06	−0.12	0.26	0.40	0.47
	(Sig.)	(0.969)	(0.832)	(0.662)	(0.336)	(0.124)	(0.066)
	Drif_NO - Drif_C (sig.)	0.884			0.523		
	Drif_NO -Drif_CR (sig.)	0.777			0.497		

*Average ± SD.* *P < 0.05, **P < 0.01.

## Discussion

### Effect of Cardiac and Respiratory Noise Removal on Medial Areas

This study aimed to clarify the effects of physiological noise on the DMN and DMN-related network, and the effect of physiological noise correction on the strength of their FC. The main findings of this study were as follows: NBS analysis identified 36 networks with significant differences in three conditions in FC matrices. *Post hoc* comparison showed no differences between the three conditions, indicating that all three conditions (Drif_NO, Drif_C, and Drif_CR) had the same networks. However, among 36 networks, FC strength of eight networks was specifically modified under physiological noise correction. FC strength increased in three networks, while FC strength decreased in five networks. In three networks, FC strength increased between the left and right superior medial frontal gyrus, between the left and right anterior cingulate gyrus, and between the right supramarginal gyrus and the right precuneus. In another five networks, FC strength was decreased in the following networks: between the left and right inferior temporal gyrus, between the right inferior temporal gyrus and the left inferior parietal gyrus, between the right inferior temporal gyrus and the left angular gyrus, between the left superior medial frontal gyrus and the left supramarginal gyrus, and between the left median cingulate gyri and the left middle cingulate gyrus.

Functional connectivity strength was increased after physiological correction was observed in the frontal medial part of the brain. It has been reported that physiological noise appears in the gray matter near the sagittal sinus (Lee et al., 2019). The superior medial frontal gyrus and the anterior cingulate gyrus are located on the medial cerebral hemisphere, close to the sagittal sinus and arteries in the cerebral hemispheres. Similarly, the parietal lobe, especially the precuneus, receives more cerebral blood flow than other brain regions and has a higher metabolic rate (Leech and Sharp, 2014). Indeed, increased FC strength around the right precuneus was also observed. Medial part of the brain may be sensitive to physiological noise, mostly cardiac noise. The other networks observed temporal regions and showed decrease in their strength of connectivity by removing physiological noise, indicating that cardiac and respiratory noise may be sensitive to BOLD signal during resting state. Connectivity strength was decreased by performing cardiac noise correction alone and cardiac and respiratory correction. Without physiological noise correction condition, head movement significantly correlated with the respiratory movement was observed in this study, which may have led to the assumption that the temporal regions were quite sensitive to respiratory activity. In addition to the movement factor, significant difference was observed between RR and *z-*value in the FC between the left inferior parietal gyrus and the right inferior temporal gyrus in all three conditions, indicating that other factors, such as respiration-related brain activity, might be involved. It is suggested that respiration-related signals might be synchronized with the BOLD signals in the DMN (Birn et al., 2006). We assume that not only movement noise related to respiration, temporal lobe, including temporal pole, superior temporal gyrus and insula, was highly correlated with anxiety related respiration changes (Masaoka and Homma, 2000), speech processing (Dien et al., 2013) and respiratory sensation (Chan et al., 2018). These factors may affect the signaling sensitivity of the rs-fMRI. How the breathing rate and depth affect the FC strength in various emotion and cognitive tasks, which influence cardiac and respiration changes, remains unknown and should be further investigated in studies measuring tidal volumes as well as CO_2_ concentrations in the body.

### Benefits of Using DRIFTER

In this study, the DRIFTER toolbox was adapted to remove the physiological noise. One of the reasons for choosing DRIFTER was that we used a nasal mask to monitor cardiac and breathing conditions in real time. The DRIFTER toolbox offers the possibility of physiological noise removal without reference signals by only defining estimated frequency ranges. In previous studies, physiological noise was also successfully removed from fMRI with long repetition time (Sarkka et al., 2012; Chang et al., 2015; Rajna et al., 2015). However, the RETROICO method, which is based on fitting a low-order Fourier basis to the data and eliminating components corresponding to cardiac rate and RR together with their harmonics, is the main approach for removing physiological noise and has been used in several studies. The phases of the cardiac and respiratory cycles are estimated from reference signals by peak detection and histogram-based methods, respectively. Although we adapted DRIFTER method to remove physiological noise, one could argue that removing variance associated with physiological noise is simply adding another low-frequency filter to modify connectivity estimates. Indeed, Chang and Glover (2009) noted that low-pass filtering appeared to exert an influence in the same direction as physiological noise correction. Differences between real-time measurement respiration and cardiac output noise and low filtering remain to be investigated in future studies.

Recently, physiological noise of respiration has been considered to be insufficient to monitor respiratory movement with the chest belt alone. It has been suggested that the evaluation of the end-tidal CO_2_ (EtCO_2_) is also important for the respiratory-related physiological noise (Bulte and Wartolowska, 2017). A study reported that the FC did not change following removal of the physiological noise based on cardiac rate or respiratory volume, whereas it was preferable to consider the physiological noise caused by EtCO_2_ (Golestani et al., 2017). The underlying mechanism may be that the BOLD signal increases because CO_2_ is a potent vasodilator. Variations in breathing depth and rate lead to alterations in arterial CO_2_ levels. For example, decreased breathing depth and/or rate lead to an increased blood flow because of accumulating CO_2_ in the blood increasing its concentration. As a result, the BOLD signal is enhanced (Birn et al., 2006; Golestani et al., 2015). In the present study, we could not evaluate the physiological noise caused by EtCO_2_. CO_2_ measurements may help to detail the influences of physiological noise on the BOLD signal and will be investigated in future research.

### Evaluating Emotions Using MRI

Various emotions change cardiac output and breathing patterns (Masaoka et al., 2014b). In individuals, these physiological outputs may also in return affect the perceived emotional levels. Large increases in emotions may be accompanied by large increases in physiological parameters. Thus, MRI measurements require careful attention regarding changes in cardiac and breathing parameters in healthy subjects, as well as patients with hyperventilation and panic disorders, leading to physiological changes. This study has some limitations. The sample size was small in this study and thus a larger sample size is needed for replication and results confirmation. Larger sample may include various types of breathing pattern, and pattern difference comparison between subjects might provide additional information. This study tested the effect of three conditions on connectivity only during the resting state; thus, it is necessary to investigate the effect of physiological noise corrections on the connectivity of the medial frontal and the posterior cingulated gyrus activated by emotional or memory tasks.

In our study, we confirmed a statistically significant effect in the medial part of the brain by removing the physiological noise. Several imaging studies have been focused on the medial prefrontal regions, anterior cingulate gyri, or inferior temporal gyrus (Bush et al., 2000; Su et al., 2015; Marek et al., 2018; Du et al., 2019; Wang et al., 2019) including the DMN (McKiernan et al., 2003; Greicius et al., 2009). These areas have reportedly major functions in emotion, meditation, relaxation, and mindfulness routines that are accompanied by manipulation of the breathing technique. Physiological parameters such as cardiac and respiratory frequencies, as well as tidal volume and EtCO_2_ measurements, may play a more important role in future research on brain–body interactions.

## Data Availability Statement

The datasets generated for this study are available on request to the corresponding author.

## Ethics Statement

The studies involving human participants were reviewed and approved by the Ethics Committee of Showa University School of Medicine. The patients/participants provided their written informed consent to participate in this study. Written informed consent was obtained from the individual(s) for the publication of any potentially identifiable images or data included in this article.

## Author Contributions

AY, YM, and MY proposed the study design and were involved in all experiments. MH, KW, and IN recruited the subjects. MY and MId performed the brain scans in these subjects. NK analyzed the data, especially the physiological noise. AY, YM, MY, and NK did additional network analysis. AY, YM, and MIz were responsible for drafting the article. All authors revised the article and provided approval for publication of the content and agreed to be accountable for all aspects of the work in ensuring that questions related to the accuracy or integrity of any part of the work are appropriately investigated and resolved.

## Conflict of Interest

The authors declare that the research was conducted in the absence of any commercial or financial relationships that could be construed as a potential conflict of interest.

## Footnotes

1 http://rfmri.org/DPARSF

2 https://www.fil.ion.ucl.ac.uk/spm/software/spm12/

3 https://www.nitrc.org/projects/nbs/
