# Supplementary material for: Heart Rate and Respiration Affect the Functional Connectivity of Default Mode Network in Resting-State Functional Magnetic Resonance Imaging
Source: Front Neurosci. 2020 Jun 30;14:631. doi: 10.3389/fnins.2020.00631 (PMC7338607; doi:10.3389/fnins.2020.00631)
Supplement: Supplementary file 1 [file Table_1.DOCX]

Supplementary Material


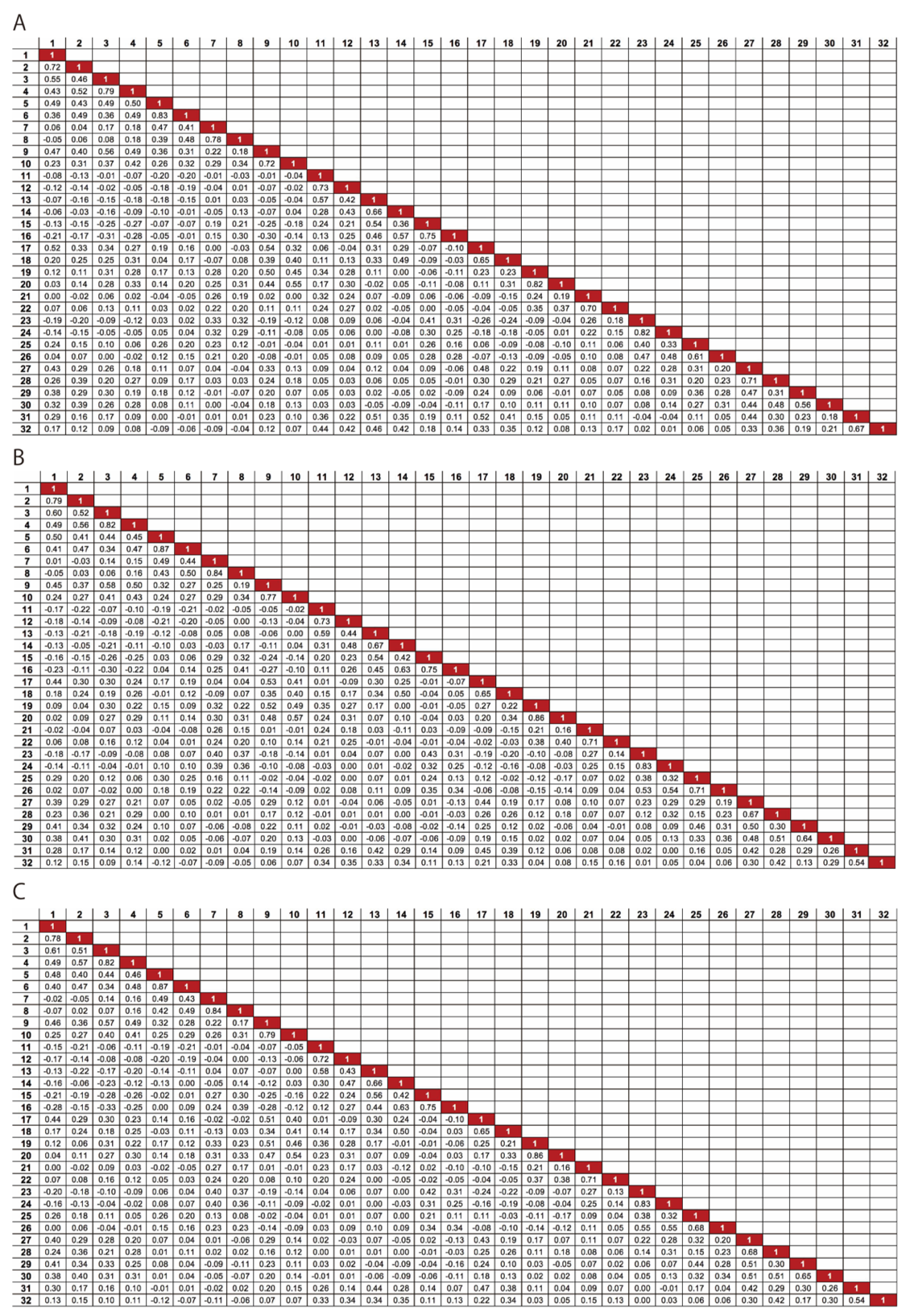


**Supplementary Figure 1.** Mean values of the correlation coefficients in the default mode network (DMN) and DMN-related brain regions in 16 subjects. (**A**) Connectivity matrix with no physiological noise correction (Drif_NO). (**B**) Connectivity matrix with corrected cardiac noise (Drif_C). (**C**) Connectivity matrix with corrected cardiac and respiratory noise signals (Drif_CR).


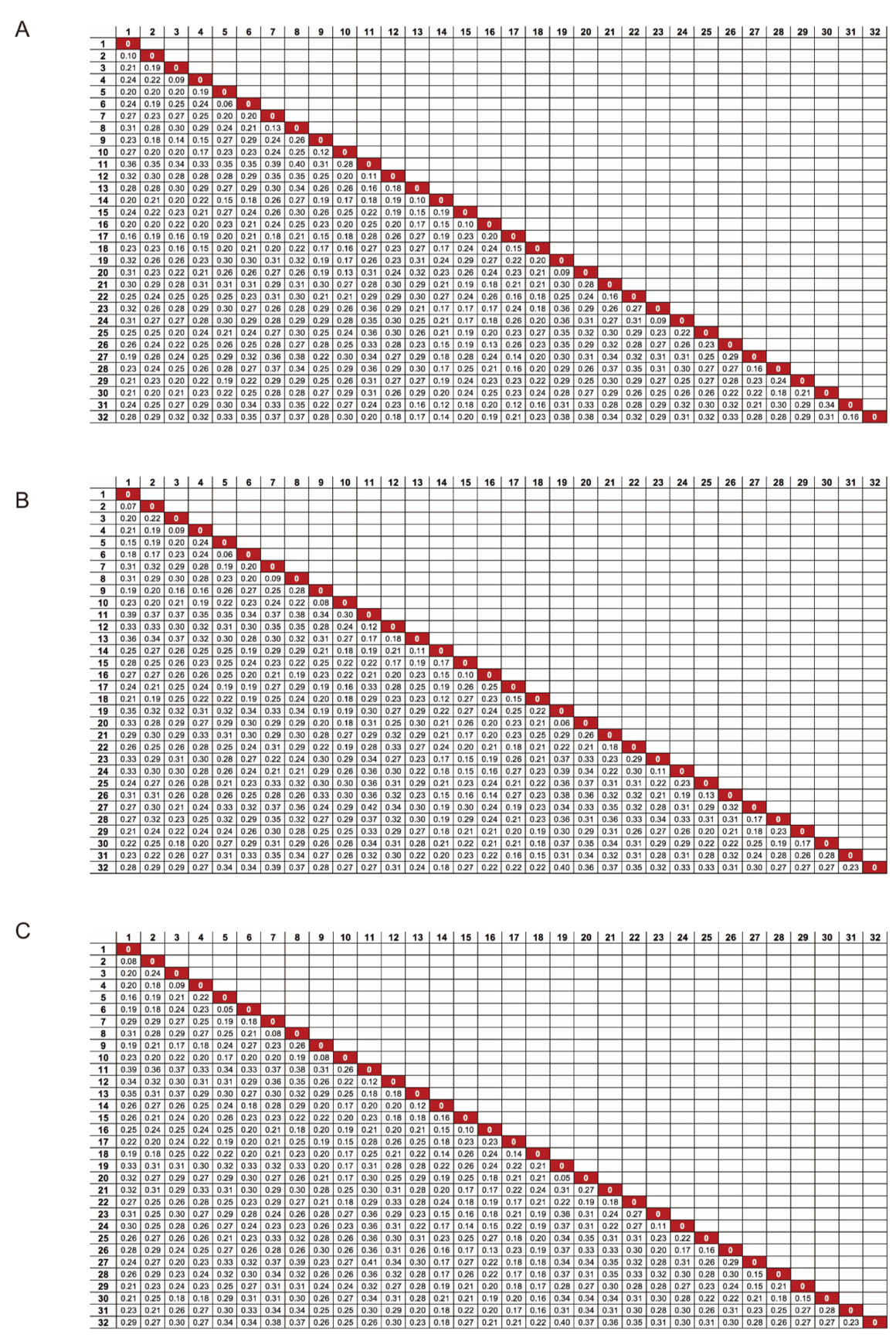


**Supplementary Figure 2.** Average of standard deviations in the default mode network (DMN) and DMN-related brain regions in 16 subjects. (**A**) Connectivity matrix with no physiological noise correction (Drif_NO). (**B**) Connectivity matrix with corrected cardiac noise (Drif_C). (**C**) Connectivity matrix with corrected cardiac and respiratory noise signals (Drif_CR).

**
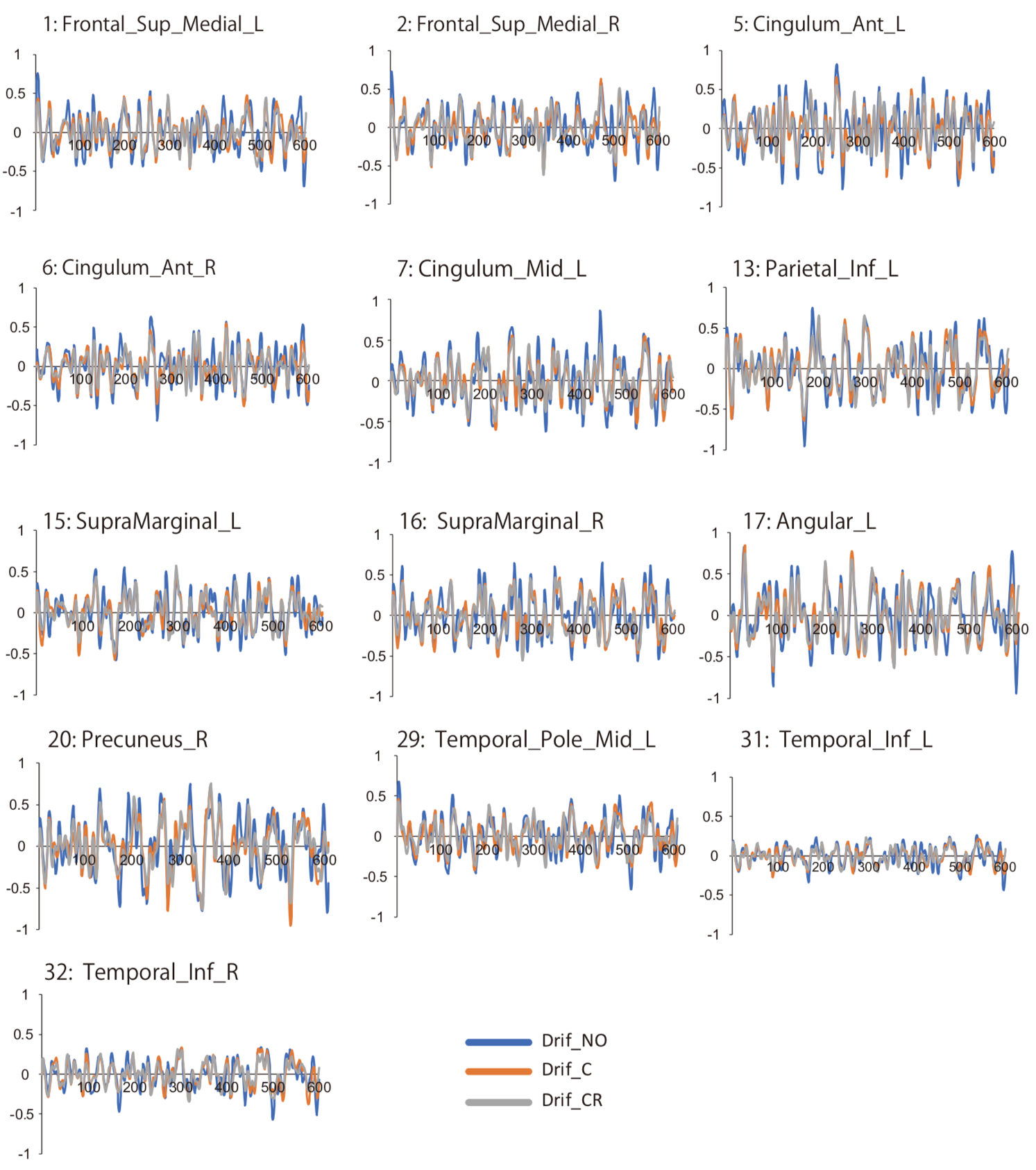
**

**Supplementary Figure 3.** Temporal changes in 600-s BOLD signals in raw data. Each node having significant difference following paired t-test and post-hoc FDR correction analysis is indicated. See Figure 4D Supplementary Table 1 and Supplementary Table 2 for more detail.

**Supplementary Table 1.** Default mode network (DMN) and DMN-related areas extracted in this study.

| node ID |  | x | y | z |
| --- | --- | --- | --- | --- |
| 1 | Superior medial frontal gyrus (left) | -6 | 49 | 31 |
| 2 | Superior medial frontal gyrus (right) | 8 | 51 | 30 |
| 3 | Superior medial orbital frontal gyrus (left) | -6 | 54 | -7 |
| 4 | Superior medial orbital frontal gyrus (right) | 7 | 52 | -7 |
| 5 | Anterior cingulate and paracingulate gyri (left) | -5 | 35 | 14 |
| 6 | Anterior cingulate and paracingulate gyri (right) | 7 | 37 | 16 |
| 7 | Median cingulate and paracingulate gyri (left) | -6 | -15 | 42 |
| 8 | Median cingulate and paracingulate gyri (right) | 7 | -9 | 40 |
| 9 | Posterior cingulate gyrus (left) | -6 | -43 | 25 |
| 10 | Posterior cingulate gyrus (right) | 6 | -42 | 22 |
| 11 | Superior parietal gyrus (left) | 24 | -60 | 59 |
| 12 | Superior parietal gyrus (right) | 25 | -59 | 62 |
| 13 | Inferior parietal gyrus (left) | -44 | -46 | 47 |
| 14 | Inferior parietal gyrus (right) | 45 | -46 | 50 |
| 15 | Supramarginal gyrus (left) | -57 | -34 | 30 |
| 16 | Supramarginal gyrus (right) | 57 | -32 | 34 |
| 17 | Angular gyrus (left) | 45 | -61 | 36 |
| 18 | Angular gyrus (right) | 45 | -60 | 39 |
| 19 | Precuneus (left) | -8 | -56 | 48 |
| 20 | Precuneus (right) | 9 | -56 | 44 |
| 21 | Paracentral lobule (left) | -9 | -25 | 70 |
| 22 | Paracentral lobule (right) | 6 | -32 | 68 |
| 23 | Superior temporal gyrus (left) | -54 | -21 | 7 |
| 24 | Superior temporal gyrus (right) | 57 | -22 | 7 |
| 25 | Superior temporal gyrus_Temporal pole (left) | -41 | 15 | -20 |
| 26 | Superior temporal gyrus_Temporal pole (right) | 47 | 15 | -17 |
| 27 | Middle temporal gyrus (left) | -57 | -34 | -2 |
| 28 | Middle temporal gyrus (right) | 56 | -37 | -1 |
| 29 | Middle temporal gyrus_Temporal pole (left) | -37 | 15 | -34 |
| 30 | Middle temporal gyrus_Temporal pole (right) | 43 | 15 | -32 |
| 31 | Inferior temporal gyrus (left) | -51 | -28 | -23 |
| 32 | Inferior temporal gyrus (right) | 53 | -31 | -22 |

**Supplementary Table 2.** The BOLD signals average in 13 nodes with significant difference. The data indicate average ± AD and *p*-value of ANOVA. See Figure 4E and Supplementry Table 1 in this table list.

|  | **Drif_NO** | **Drif_C** | **Drif_CR** | **Sig. (*p*)** |
| --- | --- | --- | --- | --- |
| 1: Superior medial frontal gyrus (left) | 0.0014 ± 0.014 | -0.0011± 0.011 | -0.0009 ± 0.009 | 0.805 |
| 2: Superior medial frontal gyrus (right) | 0.0012 ± 0.015 | -0.0013 ± 0.011 | -0.0020 ± 0.010 | 0.767 |
| 5: Anterior cingulate gyri (left) | 0.0002 ± 0.019 | -0.0005 ± 0.013 | 0.0005 ± 0.013 | 0.982 |
| 6: Anterior cingulate gyri (right) | -0.0007 ± 0.016 | -0.0006 ± 0.011 | 0.0003 ± 0.011 | 0.973 |
| 7: Median cingulate and gyri (left) | -0.0003 ± 0.015 | -0.0010 ± 0.011 | -0.0010 ± 0.010 | 0.982 |
| 13: Inferior parietal gyrus (left) | -0.0033 ± 0.011 | -0.0015 ± 0.009 | -0.0006 ± 0.010 | 0.755 |
| 15: Supramarginal gyrus (left) | -0.0004 ± 0.009 | -0.0009 ± 0.009 | 0.0000 ± 0.009 | 0.961 |
| 16: Supramarginal gyrus (right) | 0.0018 ± 0.011 | 0.0000 ± 0.010 | 0.0008 ± 0.010 | 0.895 |
| 17: Angular gyrus (left) | -0.0036 ± 0.011 | -0.0033 ± 0.010 | -0.0026 ±0.010 | 0.966 |
| 20: Precuneus (right) | -0.0040 ± 0.018 | -0.0048 ± 0.015 | -0.0047 ± 0.013 | 0.987 |
| 29: Middle temporal gyrus_Temporal pole (left) | 0.0039 ± 0.010 | 0.0002 ±0.010 | 0.0009 ± 0.009 | 0.544 |
| 31: Inferior temporal gyrus (left) | -0.0028 ± 0.006 | -0.0032 ±0.006 | -0.0023 ± 0.004 | 0.920 |
| 32: Inferior temporal gyrus (right) | -0.0034 ± 0.009 | -0.0042 ±0.008 | -0.0039 ± 0.007 | 0.966 |
